# Introducing the *Safe Brain Initiative’s EEG boot camp for anaesthesia* for standardised training on how to use the electroencephalogram for perioperative care

**DOI:** 10.1186/s12871-025-03276-8

**Published:** 2025-09-20

**Authors:** Falk von Dincklage, Janna Helfrich, Susanne Koch, Martin Soehle, Joana Berger-Estilita, Viktor Bublitz, Vincent Bonhomme, Jamie Sleigh, Gerhard Schneider, Matthias Kreuzer, Finn Radtke

**Affiliations:** 1 Department of Anaesthesia, Intensive Care, Emergency and Pain Medicine, University Medicine Greifswald, Greifswald, Germany; 2 Department of Anaesthesiology, Yale School of Medicine, New Haven, United States; 3 Department of Anaesthesiology IRS, Nykøbing F. Hospital, Nykøbing Falster, Denmark; 4 Department of Anaesthesiology, Charité - University Medicine Berlin, Berlin, Germany; 5 Department of Anaesthesiology and Intensive Care Medicine, University Hospital Bonn, Bonn, Germany; 6 Institute of Anaesthesiology and Intensive Care, Hirslanden Medical Group, SalemspitalBern, Switzerland; 7 Institute for Medical Education, University of Bern, Bern, Switzerland; 8 Centre for Health Technology and Services Research, Faculty of Medicine, University of Porto, Porto, Portugal; 9 Department of Anesthesia and Intensive Care Medicine, Liege University Hospital, Liege, Belgium; 10 Anesthesia and Perioperative Neuroscience Laboratory, GIGA-Consciousness, GIGA-Neuroscience, Liege University, Liege, Belgium; 11 Department of Anaesthesiology, Waikato Clinical Campus, University of Auckland, Hamilton, New Zealand; 12 Department of Anaesthesiology and Intensive Care, School of Medicine and Health, Technical University of Munich, Munich, Germany

**Keywords:** Electroencephalogram, Anaesthesia, Monitoring, Training

## Abstract

**Background:**

Monitoring the brain under general anaesthesia using the electroencephalogram (EEG) can help to optimise anaesthetic levels and improve patient outcomes. Therefore, it has been recommended by several societies and organisations. Yet, many clinicians only consider the processed indices, even though they are prone to interference and their information value is limited in many situations. To use EEG monitoring systems to their full potential, clinicians need to be able to integrate all information provided. Here, we introduce a structured teaching course and evaluate its effect on the participants’ knowledge and attitudes.

**Methods:**

The course contents were derived from learning goals, that we considered as required to leverage the full potential of the EEG monitoring systems. The course structure was built using several didactic tools to facilitate learning, including a high level of algorithmisation as well as tools for knowledge repetition, activation, and transfer. To investigate the effects of the course, we compared the participants’ self-ratings of their knowledge with regard to the learning goals as well as their attitudes towards using EEG monitoring before and after the course. For this purpose, we anonymously questioned the participants of one course conducted in Greifswald/Germany in December 2023.

**Results:**

The ratings of 36 participants before and after the course show that participation led to a significant improvement in knowledge throughout all learning goals (paired Wilcoxon signed-rank tests, p < 0.001 for each learning goal). Self-ratings of knowledge and competence increased across all learning goals from a mean of 1.9 before the course to 4.0 after the course, rated on Likert scales between 0 (‘No knowledge/competency’) and 5 (‘Expert knowledge/competency’). Furthermore, the attitude towards applying EEG monitoring during general anaesthesia improved significantly (paired Wilcoxon signed-rank test, p = 0.019) from 3.0 ± 1.7 to 3.8 ± 1.2 (mean ± sd), rated on a Likert scale between 0 (‘never’) and 5 (‘always’).

**Conclusions:**

We show that the course improves the participants’ self-ratings of knowledge with and attitude towards EEG monitoring. By providing teaching methods and resources with standardized contents we aim to facilitate training of the highest quality and motivating clinicians to improve anaesthesia practice, and ultimately patient outcome.

**Supplementary Information:**

The online version contains supplementary material available at 10.1186/s12871-025-03276-8.

## Background

The standard monitoring of a patient undergoing general anaesthesia focuses primarily on heart activity, circulation, respiration, and body temperature [1]. The brain, however, as the primary target organ of anaesthetics, is often not covered, although monitoring systems that incorporate processed information from the electroencephalogram (EEG) have been available for quite some time [2–6].

One of the main reasons for the neglect of EEG monitoring by anaesthesiologists is that it suffers from a poor reputation, which primarily originates from the fact that EEG monitoring during anaesthesia is often equated to processed EEG indices [7]. Thus, the shortcomings and inconsistencies of the processed EEG indices are interpreted as shortcomings and inconsistencies of EEG monitoring in general [7]. This has fostered quite a few controversial discussions about its capability and relevance, e.g. to prevent intraoperative awareness [8–10], to identify too deep levels of anaesthesia [2, 11], or to correctly deal with artefacts [12, 13]. Other publications question the EEG indices’ usefulness because of their time delay [14–16], their sensitivity to muscle activity [12, 13], or their unreliability in detecting burst suppression [17–19]. However, virtually all of these shortcomings could be avoided or mitigated if all the information available from anaesthesiologic EEG monitoring was used—- and not just the isolated EEG indices.

Due to the shortcomings of solely relying on processed EEG indices, and the potential benefits that a more comprehensive EEG interpretation might offer to improve patient outcomes, several professional bodies now recommend such interpretation as part of routine anaesthesia care [20, 21]. However, anaesthesiologists need training to interpret and understand all the available information. This includes reading the"raw"EEG trace, analysing DSAs, and understanding the limitations of processed indices. Since every currently marketed anaesthesiologic EEG monitor offers visualizations of the raw EEG trace as well as the DSAs, leveraging these requires no other equipment in comparison to only using the processed EEG index numbers, but only training. To facilitate this, several high-quality publications exist [22, 23], and the number of workshops at hospitals and conferences that focus on EEG monitoring in anaesthesiology is increasing. But the content and quality of these workshops vary widely depending on the individual organisers and speakers.

In order to improve the quality of EEG training courses, the Safe Brain Initiative (SBI)—now an ESAIC research group– developed the *SBI’s EEG boot camp for anaesthesia* as a standardised course program that focuses on teaching the fundamentals of EEG interpretation for anaesthesiologists. The course content was derived from learning goals that reflect the practical needs of clinicians to leverage the full potential of EEG monitoring systems, and the course structure utilises several didactic tools to facilitate learning, including a high level of algorithmisation as well as tools for knowledge repetition, activation, and transfer.

In this article, we aim to introduce the standardised content catalogue and educational framework of the *SBI’s EEG boot camp for anaesthesia*, and to validate its effects on the knowledge and attitudes of course participants, by comparing the participants’ self-ratings before and after a course.

## Methods

### The concept of SBI’s EEG boot camp for anaesthesia

The *SBI’s EEG boot camp for anaesthesia* was developed as part of the Safe Brain Initiative (SBI)—now an ESAIC research group—that focuses on to improving patient outcomes through patient-centred precision care (https://safebraininitiative.com/). Based on real-world evidence, SBI employs an advanced data-driven dashboard for continuous structured routine feedback to healthcare professionals. The core of the SBI is that posing the simple question *‘How are your patients doing after anaesthesia?’* can significantly improve patient care, as every clinician inherently strives to optimize quality of care. In this context, the EEG was identified as an essential tool to improve post-anaesthesia outcome. Hence, the SBI’s EEG boot camp for anaesthesia was founded in 2023 in Nykobing, Denmark. It has been iteratively developed and improved over several boot camps held in different locations, including Nykobing/Denmark (06–23), Glasgow/UK (10–23), Antalya/Turkiye (11–23), and Greifswald/Germany (12–23).

The course contents were derived from defined learning goals and then sorted into theoretical blocks that build on each other, concluding with a final block providing consolidation through practical training. In addition to theoretical and hands-on training, all learning is facilitated by providing all contents and learning goals in problem-based, algorithmic concepts (examples shown in supplement 1). With the algorithms, the participants receive clearly defined clinical strategies that they can directly incorporate into daily practice. Furthermore, learning consolidation is supported through online-based quiz rounds after each block as well as by actively abstracting the knowledge by transferring it from cases in the operating room to the intensive care unit (ICU).

### Learning goals

To define the contents for the *SBI’s EEG boot camp for anaesthesia* we first defined what knowledge we consider essential to leverage the full potential of EEG monitors. To provide motivation for using EEG monitors at all, users are required (1) to know and understand how EEG monitoring can contribute to improving patient outcomes. To enable users to actually perform EEG monitoring, they need (2) to be able to apply EEG monitoring correctly. To enable users to interpret EEG monitoring for guiding anaesthetic level, they have (3) to understand how EEG monitoring could indicate (3a) too shallow, (3b) adequate or (3c) too deep anaesthesia. To be able to be sure that recognised signs of too shallow, adequate or too deep anaesthesia are actually valid, they need (4) to be able to identify artefacts, which requires (5) to understand how EEG monitoring works (physiologically and technologically) and (6) to understand the different types of information visualisation on EEG monitors (raw EEG, spectrogram, processed indices). Moreover, as different anaesthetics manifest with distinct effects on the EEG, it is necessary for users (7) to know about the different EEG patterns induced by different substances used for anaesthesia. To avoid confusing patterns caused by nociception and insufficient dosing of analgesics with patterns of a too shallow hypnotic depth, they require (8) to be able to identify signs of nociception in the EEG. And finally, to adapt the knowledge to specific patient populations, they are required (9) to know about EEG differences in geriatric as well as in paediatric patient populations.

### Structure of the SBI’s EEG boot camp for anaesthesia

**Fig. 1:**
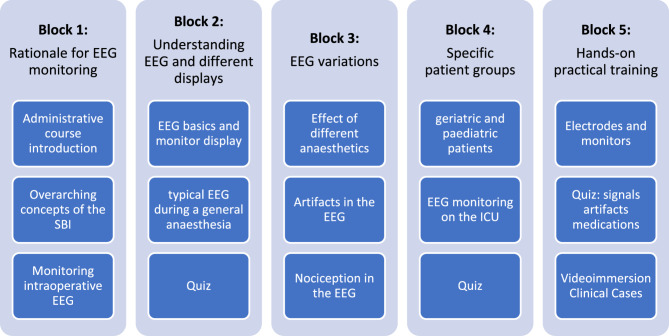
Course outline

As the order in which we logically derived the learning goals is not necessarily the best order in which to teach their contents, we restructured the course contents into four theoretical blocks that build on each other, concluding with a final block providing consolidation for training (see Fig. 1).

#### Block 1: Rationale for EEG monitoring during anaesthesia

After an administrative introduction into the course, the second part of the first block aims at putting EEG monitoring in the context of the general approach of an outcome-focused anaesthesia practice, which is the overarching concept of the Safe Brain Initiative. After this general motivation to enhance patient outcomes by optimizing patient care, the course narrows its focus to EEG monitoring. It presents specific evidence on how too deep or too shallow anaesthesia can impact outcomes and illustrates how EEG monitoring can aid in avoiding these scenarios. A key focus is on burst suppression, an EEG pattern marked by low-amplitude periods interrupted by bursts of activity. Burst suppression is recognised as a potential risk factor for the development of postoperative neurocognitive disorders [24, 25]. However, some contradictory findings [26] led to the European Society of Anaesthesiology’s ‘weak’ recommendation for EEG-based monitoring [20]. Due to the non-standardized and unreliable automated detection of burst suppression in monitoring systems [17, 19], it is crucial to include raw EEG and DSA analysis, which is a primary motivation for this course.

#### Block 2: Understanding the EEG and the different displays of the monitor

##### What does the EEG measure and how does the monitor display information?

The second block starts with one lecture providing fundamental knowledge on how the EEG is measured from a physiological, technical and practical perspective. Beginning with the physiological introduction, knowledge is provided on what the EEG actually is, including a short description of how the EEG and its synchronous activity are generated by the (cortical) neurons and how the raw EEG is abstracted into DSA and index values. After the basics of EEG physiology, in the technical introduction, we demonstrate how the raw EEG, the DSA and the indices are presented on the monitoring systems and how the display can be optimised for clinical decision-making. Concluding with the practical introduction, we teach the basics of how to properly set up anaesthesiologic EEG monitoring, including different possibilities of recording setups and covering the complete process from identifying the site for monitoring, skin preparation, proper electrode attachment, and how to maintain a good recording quality throughout a case.

##### What does EEG monitoring show during a typical general anaesthesia?

After laying the groundwork for EEG monitoring in the first lecture, the second lecture actively builds upon this knowledge by presenting the first exemplary cases. This session focuses on relatively straightforward general anaesthesia cases without complicating factors, highlighting typical EEG patterns during induction, maintenance, and recovery from anaesthesia. Additionally, it provides practical algorithms on how EEG monitoring can guide or assist at each stage.

##### Quiz to activate and repeat knowledge of blocks 1 + 2

The second block concludes with a dynamic online quiz to activate participants'knowledge. Participants answer multiple-choice questions from lectures in blocks 1 and 2 through their smartphones, using platforms (e.g. using kahoot.com). Following their submissions, the system displays the answer distribution, and the respective lecture speaker explains the correct answers. Although we present test results anonymously, participants can use nicknames for personalised feedback.

This quiz engages participants in decision-making, thereby triggers them to activate knowledge gained up to this point and reinforces crucial points through the speakers'explanations. It transitions the format from lecture-based to interactive, inviting participants to ask questions about topics from the first two blocks. This setup encourages discussions, which participants can extend into the following break for more personalised interactions.

#### Block 3: Identifying EEG variations depending on the situation

##### How do I recognise the effect of different anaesthetics in the EEG?

Since the second block has provided the basics of EEG monitoring and what to expect in a typical patient during the different phases of general anaesthesia, the third block focuses on introducing variability. The first and most important aspect of variability in EEG monitoring during general anaesthesia is the heterogeneity of EEG patterns induced by different anaesthetics. Thus, this lecture describes how typical GABAergic anaesthetics and non-GABAergic substances influence the intraoperative EEG differently and how the processed EEG indices react to these changes.

It’s highlighted that processed EEG indices, originally designed to reflect the anaesthetic level induced by GABAergic substances (notably the slowing of the EEG and the emergence of delta rhythms [27]), may lose their validity with other substances like ketamine or dexmedetomidine that produce different EEG patterns. Particularly, ketamine can cause oscillatory activity in higher frequencies (around 30 Hz), potentially increasing index values despite a deeper anaesthetic level [28], depending on the ketamine concentration [29].

Importantly, regardless of the EEG indices, all these substances create unique signatures in both the EEG and the DSA. Thus, this module educates participants on how visual analysis of the EEG, augmented by DSA, can effectively identify the effects of various anaesthetics and aid in guiding anaesthesia. This is especially relevant when processed indices may not be reliable due to the use of non-GABAergic drugs.

##### How do I recognise artefacts in the EEG?

The perioperative period, being susceptible to various disturbances, often results in physiological and technical EEG artefacts, adding further variability to measurements. Addressing this, the second lecture in this block concentrates on the most common artefacts, offering algorithms to identify them and pinpoint their sources. This lecture also explains how artefacts can result in scenarios where no index is displayed due to a contaminated EEG signal. Moreover, it details how such contaminated signals can mislead the processed EEG indices, causing them to display inaccurately high or low values [30–32].

##### How do I recognise nociception in the EEG?

Contrary to common perception, the'depth'or level of general anaesthesia is not a unidimensional phenomenon [33]. Particularly when anaesthesia involves a combination of anaesthetics and potent anti-nociceptive substances like opioids, it’s crucial to consider the nociception vs anti-nociception balance as a separate yet interconnected dimension from the hypnosis vs arousal balance [34]. Nociception and anti-nociception, primarily mediated by spinal-subcortical processes, cannot be directly monitored by cerebrocortical EEG, which focuses on the hypnotic component of anaesthesia. However, nociception, acting as a potent arousing stimulus, can be indirectly reflected in EEG changes such as an alpha dropout [35, 36], a delta arousal [37, 38] or a beta activation [5, 39, 40].

This lecture introduces participants to the multidimensional nature of anaesthesia. It covers how nociceptive processing continues during general anaesthesia, its impact on patient outcomes, and how nociception can be spotted in the raw EEG, the DSA, and the processed EEG indices. The lecture also provides clinical algorithms to distinguish responses from those due to nociception from those due to non-nociceptive arousal.

#### Block 4: Identifying EEG variations depending on patient characteristics

##### What is different in geriatric and paediatric patient populations?

The fourth block delves into additional factors that introduce variability in EEG monitoring, emphasising that substances and a patient's age can affect the EEG under anaesthesia, thereby influencing processed EEG data [41–43]. In this block's first lecture, age-specific changes observable in the EEG monitoring of geriatric and paediatric patients are discussed. While the primary focus of this boot camp is on adult patients, there's a brief exploration of EEG differences in infants before shifting to age-induced EEG changes in adults. The lecture highlights that most monitoring systems do not account for age in index calculation. Therefore, it guides on how to interpret the index, raw EEG, and DSA, particularly in the geriatric population and patients with a frail brain [44, 45].

##### How can I transfer my expertise from the operating room to sedation in the ICU?

To synthesise and apply the knowledge acquired so far, this discussion block presents exemplary ICU cases. These cases are designed to explore the potential application of anaesthesiologic EEG monitoring to manage ICU patients'sedation levels. This shift from the operating room to the ICU context and from general anaesthesia to ICU sedation challenges participants to abstract and transfer their knowledge, aiding consolidation.

##### Quiz to activate and repeat knowledge of blocks 3 + 4

The fourth block includes a second interactive online quiz for knowledge activation, similar to the first. Multiple-choice questions from the lectures in blocks 3 and 4 are presented, and participants submit their answers through their smartphones. The respective speakers then explain the correct answers, mirroring the approach of the first quiz.

#### Block 5: Repetition for consolidation and practical training

The last teaching block of the course provides further repetition of the content, but in a practical hands-on setting. To reduce the size of the complete group of participants for practical hands-on training, it is split into three sub-groups, which rotate through three different stations:

##### Split-group Station 1: Placing the electrodes and operating the monitors

This hands-on station is dedicated to teaching practical skills in setting up and operating EEG monitors. The lecturers first demonstrate various recording setups, encompassing the entire process from selecting the monitoring site, preparing the skin appropriately, and attaching the electrodes properly. This includes showcasing alternative setups like mandibular or nasal montages. Participants are also guided through the different monitor settings, such as filter, time, and amplitude adjustments, and how to access various types of information like impedance, signal quality, raw EEG, DSA, and indices. After the demonstration, participants can practice with monitors (ideally from all available brands) on each other, allowing them to experiment with the different equipment.

##### Split-group Station 2: Quiz about signals, artefacts and medication signatures

The second station offers a playful quiz integrating different aspects of EEG monitoring, focusing on distinguishing between various signals, artefacts, and anaesthetic substance signatures. This interactive activity consolidates learning from the theoretical blocks by challenging participants to apply their knowledge fun and engagingly.

##### Split-group Station 3: Video immersion into clinical cases

In this station, the focus shifts to understanding the role of EEG monitoring within the broader clinical context of general anaesthesia. Participants watch complete anaesthesia cases, from pre-anaesthesia preparation to post-anaesthesia care, captured through multiple cameras. These videos showcase the complexity of clinical situations, highlighting how to incorporate EEG monitoring into specific clinical processes without detracting from other vital procedures and monitors. This immersive experience helps participants to appreciate the practical application of EEG monitoring in a real-world setting.

#### Block 6: Debriefing

The course concludes with a debriefing block, mirroring the administrative introduction at its start. This final segment aims to address any remaining questions, gather oral feedback, and collect written evaluations from the participants. Additionally, participants are guided on how to continue expanding their knowledge post-course, including directions on where to find further information and seek guidance for specific queries.

All participants will be granted access to a dedicated website, enabling them to share specific questions and interesting cases with their peers and course instructors. Similar to the course introduction, special attention is given to allotting sufficient time for this debriefing. This ensures that participants leave the course feeling well-informed and oriented, significantly influencing their overall attitude towards the course, its content, and learning outcomes. Consequently, the duration of the debriefing is planned to be equal to that of the split-group stations in the previous block, ensuring a comprehensive and satisfying course conclusion.

### Investigation of the effects of the course

To assess the effect of the *SBI’s EEG boot camp for anaesthesia* on the participants’ knowledge of the defined learning goals and their attitudes toward incorporating EEG monitoring into routine anaesthesia practice, we compared participants’ self-ratings on Likert scales (0–5) before and after the course (questionnaire shown in supplement 2). For this purpose, we obtained anonymous ratings of the participants before and after the 2023 course, held in Greifswald, Germany. As participation in the evaluation was voluntary for the participants and fully anonymous, the need for a formal ethics approval and written informed consent was waived by the local ethics committee (Ethikkommission– Universitätsmedizin Greifswald).

A formal power analysis was not performed due to the exploratory nature of the investigation regarding the size of the effects of the course. Pre- and post-course ratings were compared using the paired Wilcoxon signed-rank test, with a significance threshold set at p < 0.05.

## Results

Of the overall number of 37 participants, 36 completed the full pre-course and the full post-course questionnaires. The demographic data of these are shown in Table 1.

**Table 1:**
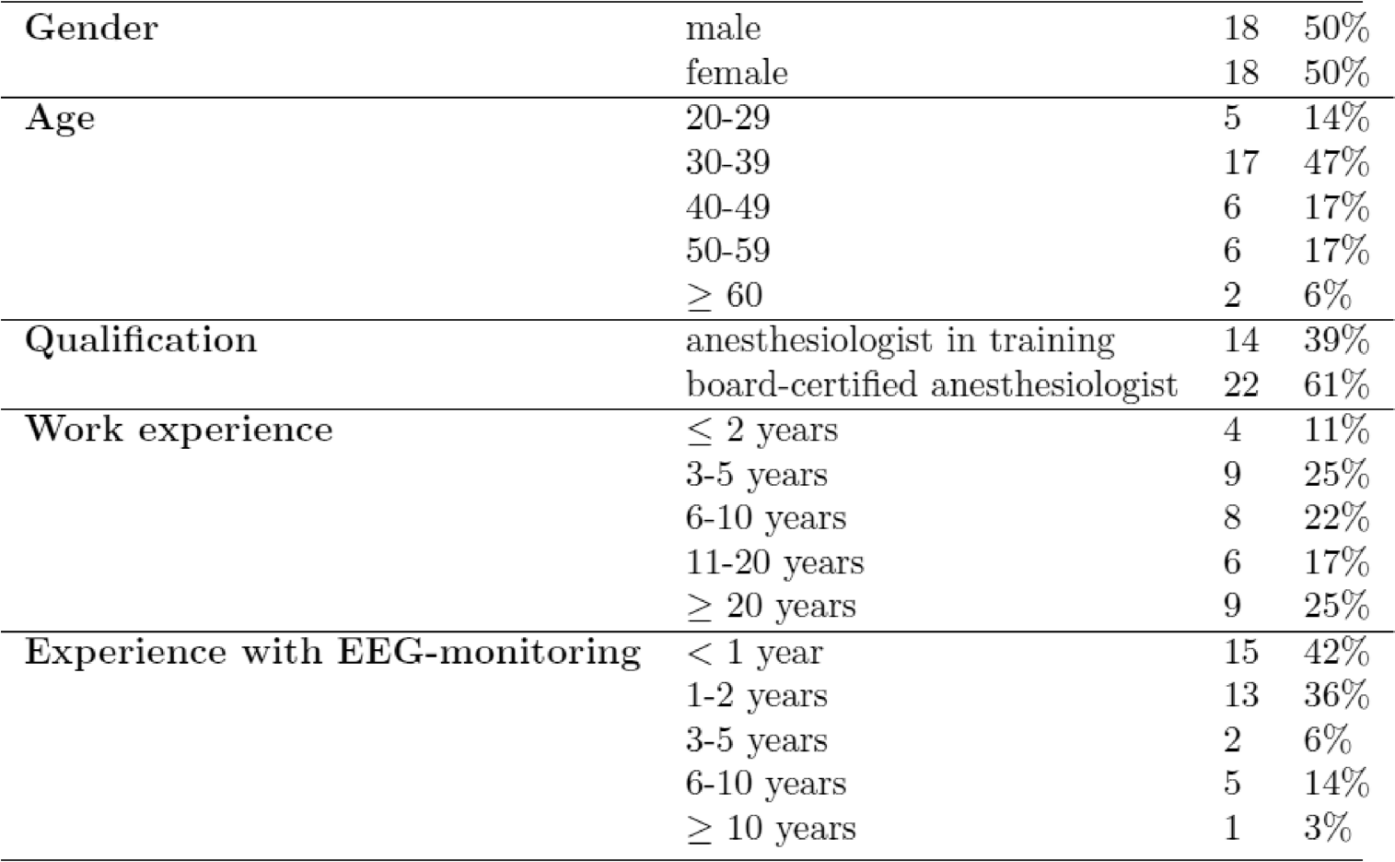
Demographic data of validation group

### Ratings of the course, its components and length

The participants rated the overall course predominantly as ‘very good’ with a mean score of 4.6 on a Likert scale between 0 (‘very bad’) and 5 (‘very good’). Using the same scale, the mean ratings of the different components of the course were 4.5 for the lectures, 4.0 for the quizzes and 4.0 for the split groups. Based on these ratings, we consider the overall structure of the course and the contributing role of each component as confirmed.

The rating of the overall length of the boot camp on a Likert scale between 1 (‘Far too short’), 3 (‘Exactly right’), and 5 (‘Far too long’) revealed two distinct groups as 19 participants gave a rating at ‘Exactly right’ and 14 participants gave a rating at ‘Far too long’. Since the course for which these ratings were obtained was a full 8-h course, we interpret these results as confirmation for our approach to offer two different versions of the course, either a full 8-h or an abbreviated 4-h version. These different versions offer opportunities to satisfy the learning needs of both groups of clinicians: those that want a quick overview and those that prefer more time to reach their personal level of comfort with EEG monitoring in anaesthesia.

### Improvement in learning goals and change in attitude

The comparison of ratings before and after the course show that participation in the *SBI’s EEG boot camp for anaesthesia* leads to a significant improvement in knowledge throughout all learning goals (paired Wilcoxon signed-rank tests, p < 0.001 for each learning goal). Self-ratings of the participants’ own knowledge and competence across all learning goals increased from a mean of 1.9 before the course to 4.0 after the course, rated on Likert scales between 0 (‘No knowledge/competency’) and 5 (‘Expert knowledge/competency’). The pre-course and post-course values for each of the learning goals are shown in Table 2.

**Table 2:**
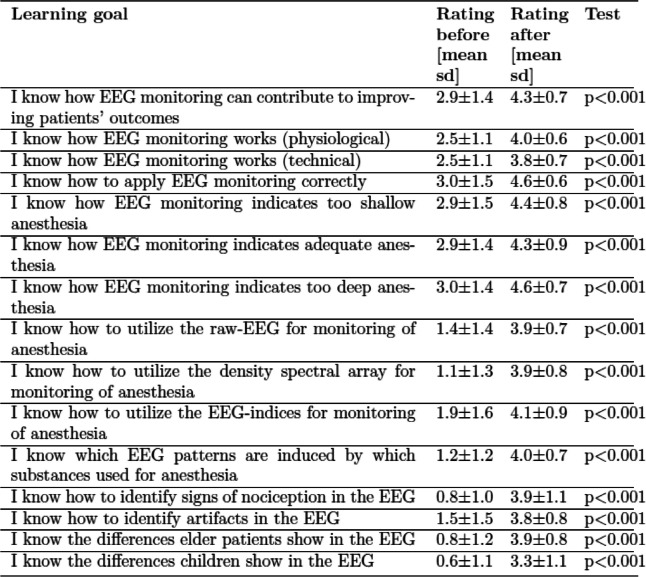
Improvement in learning goals

Furthermore, our results also show a significant improvement in attitude towards applying EEG monitoring during general anaesthesia from 3.0 ± 1.7 to 3.8 ± 1.2 (mean ± sd) on a Likert scale between 0 (‘never’) and 5 (‘always’; paired Wilcoxon signed-rank test, p = 0.019).

## Discussion

Our data shows that the here presented *SBI’s EEG boot camp for anaesthesia* improves the participants’ self-ratings of knowledge with regard to the learning goals and attitude towards including EEG monitoring in their daily anaesthesia practice.

Of course, the here presented data can only be regarded as a first, exploratory approach towards validating the effects of this course concept. Our assessment has been performed on only a relatively small sample of participants from a single centre, thus external validity is limited. Also, as this was one of the first courses, a selection bias inherent in the participant group can be expected. Since attendance at the EEG boot camp was voluntary, many participants were likely already interested in EEG monitoring and motivated to integrate it into their clinical practice, influencing both baseline attitudes and responsiveness to the course content.

Regarding internal validity, our data are based on self-reported knowledge and attitude ratings rather than objective knowledge testing or behavioural observation, which limits the strength of our conclusions regarding actual competency gains. Especially as the meaningfulness of self-ratings of attitudes is inherently limited, since it is unclear what size of an increase would be related to a change in behaviour. E.g. it remains unclear whether an increase in attitude toward EEG monitoring from 3.0 ± 1.7 to 3.8 ± 1.2 would mean that participants apply EEG monitoring more often or whether this difference is statistically significant, but clinically irrelevant.

Therefore, while our data suggests that participants perceived a meaningful improvement in their knowledge and attitudes, these perceptions cannot be used to formally validate the course content or its clinical utility. The reported results could be regarded as a necessary first step towards a structured and pedagogically sound educational intervention trial. But ultimately, the merit of the course has to be measured by its impact on clinical practice and patient outcomes in longitudinal assessments.

### What sets the SBI’s EEG boot camp apart from other ways of learning about EEG monitoring?

#### Algorithmisation

The SBI’s EEG Boot Camp for Anesthesia emphasises the immediate practical application of learned content through dedicated algorithms. Each educational module is designed to present these algorithms in a clear, easy-to-understand manner. This approach equips participants not just with theoretical knowledge but also with the confidence to integrate this new understanding into their clinical practice. We've developed acronyms like ShARP (Shape, Amplitude, Rhythm, Point in time) for artefact detection and identification to aid memory. For instance, a'spiky'shape with an amplitude similar to the EEG signal, rhythmic with one or two spikes per second, observed at any point, might indicate an electrocardiography (ECG) artifact.

#### Simplification

Focusing the contents on clinical applicability and algorithms also means that it is necessary to simplify the content, which is not always an easy task for researchers acting as teachers. Thus, many educational courses on EEG lose their participants by providing information on a too detailed level, when the expert speakers try to use their scientific presentations for educational purposes. Our course avoids overwhelming clinicians with the latest scientific minutiae, instead providing essential information for safe EEG monitoring to improve patient outcomes. The rationale can be illustrated by comparing EEG training with resuscitation training. Here, it is obvious that a basic resuscitation training course does not focus on discussing in detail what the latest findings are on a topic like why the blood actually flows when chest compressions are performed: is it rather the direct cardiac compression (‘cardiac pump theory’) or is it rather caused by changes in intrathoracic pressure (‘thoracic pump theory’) [46]? Instead, the focus of basic resuscitation training is purely on how to safely and correctly perform the procedure. In many EEG training courses, this distinction between the ever-increasing amount of scientific knowledge and the understanding necessary for safe clinical application is not made, thus overwhelming purely clinically focused participants.

#### Repetition, activation and transfer

In addition to content adjustment and restructuring for clinical application, the SBI’s EEG boot camp for anaesthesia employs a variety of didactic tools to optimise learning, focusing on repetition, activation, and active transfer. Repetition is varied across lectures, quizzes, and hands-on training. Activation involves engaging participants in applying their knowledge, like in quiz sessions using digital platforms. Active knowledge transfer requires participants to adapt what they've learned to different contexts, such as transferring knowledge from the operating room to the ICU setting. This combination of didactic methods and content tailored for clinical application positions the SBI’s EEG boot camp for anaesthesia as a state-of-the-art educational program for anaesthesiologists in EEG monitoring.

### The long-term goal

The purpose of this article is to present our approach to systematic EEG training for anaesthesiologists, in order to start a larger process of consolidation and harmonisation of course contents and training methods. The long-term goal is to align EEG training courses all over the world by providing teaching resources with standardised contents and teaching methods, similar to other formats such as in resuscitation training. By these means, training is facilitated, leading to motivated clinicians and improved anaesthesia practice, with ultimately improved patient outcomes. To achieve this, we need to establish an international and global structure providing uniform resources, but we also need local specialists to adapt the teaching resources to local needs and provide regional training courses. In the ideal case, SBI’s EEG boot camps are taught by local experts, supported by specialists already experienced with the format. Thus, we would like to invite and motivate as many peers as possible to participate as active or passive partners to further improve EEG training. We are happy to share our teaching resources free of charge and assist in setting up an *SBI’s EEG boot camp for anaesthesia* everywhere. Naturally, we are also looking to further harmonize contents and methods as well as to further improve the teaching resources by incorporating any feedback.

In order to provide an organisational structure within the ESAIC Safe Brain Initiative, we have established an advisory board of experts, who will regularly assess the SBI’s EEG boot camp structure and the educational contents, as well as a global organisation team that is responsible for coordinating SBI’s EEG boot camps and administrating the teaching resources.

To account for regional differences, we are forming regional chapters to coordinate SBI’s EEG boot camps within their regions. The most important task besides event organisation is to update and maintain the educational content. Thus, the regional groups will provide feedback to the global group at least every year to adjust the teaching resources. To enable information exchange, we are setting up a protected site, where the educational content is stored and accessible to all group members and teachers, as well as a discussion forum where former participants can ask questions or give feedback.

## Conclusion

In this article, we present the ‘SBI’s EEG boot camp for anaesthesia’ as a systematic approach of teaching an outcome-oriented use of perioperative EEG monitoring. By focusing course content on defined learning goals that truly help clinicians, a high level of algorithmisation and the utilisation of several didactic tools, we believe in presenting a new state-of-the-art EEG training for anaesthesiologists. Our data shows that the here presented course improves the participants’ self-ratings of knowledge with regard to the learning goals and attitude towards including EEG monitoring in their daily anaesthesia practice. In order to achieve the long-term goal of standardised high-quality EEG training courses worldwide, we invite all interested colleagues to collaborate so that together we will motivate clinicians worldwide to incorporate EEG monitoring into their daily practice to improve patient outcomes.

## Supplementary Information

Supplementary Material 1

Supplementary Material 2

## Data Availability

Anonymized data are available upon reasonable request.
