# Supplementary material for: Introducing the Safe Brain Initiative’s EEG boot camp for anaesthesia for standardised training on how to use the electroencephalogram for perioperative care
Source: BMC Anesthesiol. 2025 Sep 20;25:449. doi: 10.1186/s12871-025-03276-8 (PMC12449795; doi:10.1186/s12871-025-03276-8)

Supplement 1

**Exemplary Algorithms from the Course**

EEG-Preparation:

**CAChE: - C**lean the skin **- A**ttach the electrodes **- C**heck the electrodes **- E**valuate the settings

Interpretation of unexpected Signatures:

Get an **IDEA: I**ndex → **D**SA → (raw) **E**EG → **A**rtefacts?

- **Check the index** → Does it fit to what you would expect from the applied drugs and doses ?
- **Check the density spectral array**→ Does it fit to the level of anesthesia reflected by the index ?
- **Check raw EEG** → Can you identify any artefacts ?
  Be SHARP

Identifying EEG-Artifacts

- **ShARP = Sh**ape (spiky vs. slow) – **A**mplitude (compared to the EEG trace) – **R**hythm (Frequency and Regularity)– **P**oint in time (When does it occur? / Context)

Procedure in case of potential nociception:


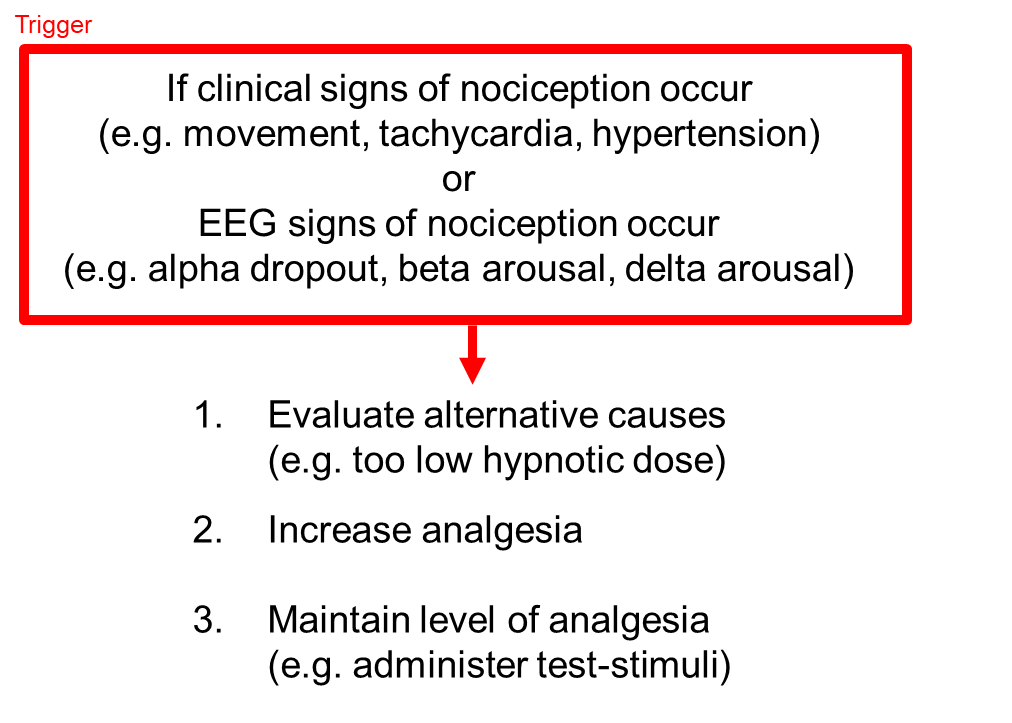

Supplement: Supplementary file 1 — Supplementary Material 1 [file 12871_2025_3276_MOESM1_ESM.docx]
