# Supplementary material for: Introducing the Safe Brain Initiative’s EEG boot camp for anaesthesia for standardised training on how to use the electroencephalogram for perioperative care
Source: BMC Anesthesiol. 2025 Sep 20;25:449. doi: 10.1186/s12871-025-03276-8 (PMC12449795; doi:10.1186/s12871-025-03276-8)
Supplement: Supplementary file 2 — Supplementary Material 2 [file 12871_2025_3276_MOESM2_ESM.docx]

Supplement 2

**EEG in Anesthesia -Bootcamp – Evaluation Before the Course**

1. You are …

○ Female
○ Male
○ Diverse

2. How old are you?

○ Younger than 20 years
○ 20–29 years
○ 30–39 years
○ 40–49 years
○ 50–59 years
○ 60 years or older

3. What is your professional group?

○ Nurse without specialization
○ Specialized nurse
○ Medical doctor without specialist qualification
○ Medical doctor with specialist qualification

4. How many years of professional experience do you have in this group?

○ Less than 1 year
○ 1–2 years
○ 3–5 years
○ 6–10 years
○ 11–20 years
○ More than 20 years

5. How many years of experience do you have with EEG monitoring?

○ No experience
○ Less than 1 year
○ 1–2 years
○ 3–5 years
○ 6–10 years
○ More than 10 years

6. What level of care does your hospital provide?

○ Non-university hospital with basic and standard care
○ Non-university hospital with specialized care
○ Non-university hospital with maximum care
○ University hospital

**Before the Course**

**Knowledge Assessment**

Please rate your knowledge about EEG monitoring in anesthesia (0 "no knowledge" to 5 "expert").

|  | **0** | **1** | **2** | **3** | **4** | **5** |
| --- | --- | --- | --- | --- | --- | --- |
| 7. I know how EEG monitoring can contribute to improving patients’ outcomes. |  |  |  |  |  |  |
| 8. I know how EEG monitoring works (physiological). |  |  |  |  |  |  |
| 9. I know how EEG monitoring works (technical). |  |  |  |  |  |  |
| 10. I know how to apply EEG monitoring correctly. |  |  |  |  |  |  |
| 11. I know how EEG monitoring indicates too shallow anesthesia. |  |  |  |  |  |  |
| 12. I know how EEG monitoring indicates adequate anesthesia. |  |  |  |  |  |  |
| 13. I know how EEG monitoring indicates too deep anesthesia. |  |  |  |  |  |  |
| 14. I know how to utilize the raw-EEG for monitoring of anesthesia. |  |  |  |  |  |  |
| 15. I know how to utilize the density spectral array for monitoring of anesthesia. |  |  |  |  |  |  |
| 16. I know how to utilize the EEG-indices for monitoring of anesthesia. |  |  |  |  |  |  |
| 17. I know which EEG patterns are induced by which substances used for anesthesia. |  |  |  |  |  |  |
| 18. I know how to identify signs of nociception in the EEG. |  |  |  |  |  |  |
| 19. I know how to identify artifacts in the EEG. |  |  |  |  |  |  |
| 20. I know the differences elder patients show in the EEG. |  |  |  |  |  |  |
| 21. I know the differences children show in the EEG. |  |  |  |  |  |  |

**EEG Monitoring Usage Assessment**

Please rate the following aspects of EEG monitoring (0 "not at all" to 5 "very much/always").

|  | **0** | **1** | **2** | **3** | **4** | **5** |
| --- | --- | --- | --- | --- | --- | --- |
| 22. How useful do you consider EEG monitoring for assessing peri- and intraoperative anesthesia depth? |  |  |  |  |  |  |
| 23. How time-consuming do you consider EEG monitoring for assessing peri- and intraoperative anesthesia depth? |  |  |  |  |  |  |
| 24. How often do you have access to EEG monitoring devices for peri- and intraoperative anesthesia depth assessment? |  |  |  |  |  |  |
| 25. How regularly have you used EEG monitoring for assessing peri- and intraoperative anesthesia depth so far? |  |  |  |  |  |  |
| 26. How regularly do you plan to use EEG monitoring for assessing peri- and intraoperative anesthesia depth in the future? |  |  |  |  |  |  |

**After the Course**

**Knowledge Assessment**

Please rate your knowledge about EEG monitoring in anesthesia (0 "no knowledge" to 5 "expert").

|  | **0** | **1** | **2** | **3** | **4** | **5** |
| --- | --- | --- | --- | --- | --- | --- |
| 27. I know how EEG monitoring can contribute to improving patients’ outcomes. |  |  |  |  |  |  |
| 28. I know how EEG monitoring works (physiological). |  |  |  |  |  |  |
| 29. I know how EEG monitoring works (technical). |  |  |  |  |  |  |
| 30. I know how to apply EEG monitoring correctly. |  |  |  |  |  |  |
| 31. I know how EEG monitoring indicates too shallow anesthesia. |  |  |  |  |  |  |
| 32. I know how EEG monitoring indicates adequate anesthesia. |  |  |  |  |  |  |
| 33. I know how EEG monitoring indicates too deep anesthesia. |  |  |  |  |  |  |
| 34. I know how to utilize the raw-EEG for monitoring of anesthesia. |  |  |  |  |  |  |
| 35. I know how to utilize the density spectral array for monitoring of anesthesia. |  |  |  |  |  |  |
| 36. I know how to utilize the EEG-indices for monitoring of anesthesia. |  |  |  |  |  |  |
| 37. I know which EEG patterns are induced by which substances used for anesthesia. |  |  |  |  |  |  |
| 38. I know how to identify signs of nociception in the EEG. |  |  |  |  |  |  |
| 39. I know how to identify artifacts in the EEG. |  |  |  |  |  |  |
| 40. I know the differences elder patients show in the EEG. |  |  |  |  |  |  |
| 41. I know the differences children show in the EEG. |  |  |  |  |  |  |

**EEG Monitoring Usage Assessment**

Please rate the following aspects of EEG monitoring (0 "not at all" to 5 "very much/always").

|  | **0** | **1** | **2** | **3** | **4** | **5** |
| --- | --- | --- | --- | --- | --- | --- |
| 42. How useful do you consider EEG monitoring for assessing peri- and intraoperative anesthesia depth? |  |  |  |  |  |  |
| 43. How time-consuming do you consider EEG monitoring for assessing peri- and intraoperative anesthesia depth? |  |  |  |  |  |  |
| 44. How often do you have access to EEG monitoring devices for peri- and intraoperative anesthesia depth assessment? |  |  |  |  |  |  |
| 45. How regularly have you used EEG monitoring for assessing peri- and intraoperative anesthesia depth so far? |  |  |  |  |  |  |
| 46. How regularly do you plan to use EEG monitoring for assessing peri- and intraoperative anesthesia depth in the future? |  |  |  |  |  |  |

**Final Feedback and Evaluation of the EEG Bootcamp**

What would need to change for you to use EEG monitoring more frequently in the future?

|  |
| --- |

Please evaluate the following qualitative content aspects of the EEG Bootcamp (0 "very poor" to 5 "very good"):

|  | **0** | **1** | **2** | **3** | **4** | **5** |
| --- | --- | --- | --- | --- | --- | --- |
| 47. How do you rate the EEG Bootcamp overall? |  |  |  |  |  |  |
| 48. How do you rate the lectures of the EEG Bootcamp? |  |  |  |  |  |  |
| 49. How do you rate the quiz parts of the EEG Bootcamp? |  |  |  |  |  |  |
| 50. How do you rate the practical group sessions of the EEG Bootcamp? |  |  |  |  |  |  |

Please evaluate the following timing aspects of the EEG Bootcamp (1 "far too short", 3 "just right", 5 "far too long"):

|  | **0** | **1** | **2** | **3** | **4** | **5** |
| --- | --- | --- | --- | --- | --- | --- |
| 51. How do you rate the overall duration of the EEG Bootcamp? |  |  |  |  |  |  |
| 52. How do you rate the time allocated to lectures? |  |  |  |  |  |  |
| 53. How do you rate the time allocated to the quiz parts? |  |  |  |  |  |  |
| 54. How do you rate the time allocated to the practical group sessions? |  |  |  |  |  |  |

What should we improve in the course? / What did you particularly like?

|  |
| --- |
